# Pregnancy Induced Hypertension among Pregnant Women Delivering in a Tertiary Care Hospital: A Descriptive Cross-sectional Study

**DOI:** 10.31729/jnma.6392

**Published:** 2021-12-31

**Authors:** Taniya Thapa, Sabita Sharma, Dipa Sigdel, Kalpana Silwai, Alisha Joshi

**Affiliations:** 1 Department of Women's Health and Development, Chitwan Medical College, Bharatpur, Chiwan, Nepal; 2 Department of Pediatric Nursing, Chitwan Medical College, Bharatpur, Chitwan, Nepal; 3 Department of Psychiatric Nursing, Chitwan Medical College, Bharatpur, Chitwan, Nepal

**Keywords:** *gestation*, *pregnancy induced hypertension*, *women*

## Abstract

**Introduction:**

Pregnancy Induced Hypertension is a major health issue with limited studies conducted so far in Chitwan, Nepal regarding adverse perinatal outcomes in obstetric population. This study aimed to find prevalence of pregnancy induced hypertension among pregnant women delivering in a tertiary care hospital.

**Methods:**

A descriptive cross-sectional study was conducted in a teaching hospital of Chitwan, Nepal during the study period of six months from 15th Jan 2019-16th July 2019 after getting ethical approval from Chitwan Medical College-Institutional Review Committee (Reference number-2075/076042). Women were selected via convenience sampling technique. Face to face interview was conducted to collect socio-demographic and obstetric data whereas, data related to the fetomaternal outcomes were obtained from patient charts and delivery record books. Statistical Package for Social Sciences version 20 was used for data analysis. Point estimate at 95% confidence interval was calculated along with frequency and proportion for binary data.

**Results:**

The prevalence of pregnancy induced hypertension was found to be 91 (6.43%) (3.839.03 at 95% Confidence Interval) representing 71 (78.1%), 12 (13.2%), and 8 (8.7%) as gestational hypertension, preeclampsia and eclampsia respectively.

**Conclusions:**

The burden of pregnancy induced hypertension was found quite higher as compared to other similar studies done in Nepal. Gestational hypertension was most common type.

## INTRODUCTION

Pregnancy Induced Hypertension (PIH) is one of the commonest causes of both maternal^1^ as well as neonatal mortality^2^ and morbidity that develops as a result of pregnancy and generally regresses after delivery, affecting about 5-8 % of pregnant women, moreover 5-22% of pregnancies especially in developing countries.^3^

The mortality is closely associated with the severity of hypertension as 22% of maternal deaths accounts only due to eclampsia in Nepal,^4^ further leads to the frequency of induced labor, fetal growth restriction, admission to neonatal intensive care unit^5^ and even high incidence on those having the previous history of PIH.^6^

With the advancement in maternity services, decline in the incidence of PIH is seen, but still contributes highly to poor maternal and fetal outcome.^7^

There are very few studies on PIH and its' outcome in Nepal. Thus, this study aimed to find the prevalence of PIH among pregnant women delivering at a tertiary care hospital.

## METHODS

A descriptive cross-sectional study was conducted in the Department of Obstetrics and Gynaecology at Chitwan Medical College Teaching Hospital, Chitwan, Nepal, and data were collected during the period of six months from 15th Jan 2019-16th July 2019. Chitwan Medical College is considered as one of the referral centre for maternity and newborn cases of province 3 providing free delivery services in co-ordination with the safe motherhood program under the Government of Nepal (GoN). Pregnant women giving consent and having complete data were included in this study Women with chronic hypertension, chronic renal disease, cardiac disease, diabetes mellitus, and patient refusal as well as incomplete data were excluded were excluded.

Ethical approval was obtained from Chitwan Medical College Institutional Review Committee (CMC-IRC) with reference no-2075/076042 and permission from the hospital side as well as the concerned department was taken prior to the study. Convenience sampling technique was used.

The sample size was calculated by using the formula

n = Z^2^ × p × q / e^2^

  = 1.96^2^ × 0.5 × (1-0.5) / (0.03)^2^

  = 1068

Where,

n= required sample size
Z= 1.96 at 95% of Confidence Interval (CI)
p= prevalence taken as 50% for maximum sample size
q= 1-p
e= margin of error, 3%

Adding 10% for the non-response rate, we get a sample size of 1178. A sample size of 1415 women meeting the selection criteria for the study was included. Before data collection, verbal informed consent was taken from each pregnant woman with PIH admitted for delivery. The dignity of the respondent was secured by giving the right to reject or discontinue the study at any time. Confidentiality of the information was maintained by not disclosing the information with others as well as the data was merely used for the study purpose and was destroyed after the completion of the study.

Face to face interview was conducted in separate corner of prenatal ward with pregnant women prior delivery to collect the data regarding socio-demographic and obstetric factors using structured questionnaire. Further, data related to the maternal and fetal outcomes were obtained from patient charts and delivery record book and was all noted in preformed Performa. Diagnosis of the cases and its types (Gestational hypertension, Preeclampsia and Eclampsia) was confirmed according to patient's history, physical examination and deranged PIH profile followed by medical diagnosis by Obstetrics/ Gynecology specialists.

Statistical Package for Social Sciences version 20 and Microsoft Excel were used for data analysis. Point estimate at 95% Confidence Interval was calculated along with frequency and proportion for binary data.

## RESULTS

Out of 1415 women studied, the prevalence of pregnancy induced hypertension was found to be 91 (6.43%) (3.83-9.03 at 95% Confidence Interval) representing 71 (78.1%), 12 (13.2%) and 8 (8.7%) as gestational hypertension, preeclampsia and eclampsia respectively. The mean age of the patients with pregnancy induced hypertension was found to be 25.79±5.59 (Mean±SD) (Table 1).

**Table 1:** Cross tabulation on pregnancy induced hypertension and different variables of pregnant women (n = 91).

Variables	Pregnancy induced hypertension			Total
	GTN n (%) (n=71)	Preeclampsia n (%) (n=12)	Eclampsia n (%) (n=8)	n (%)
Age group (yrs)				
<21	14 (19.7)	0 (0)	2 (25)	16 (17.6)
21-30	42 (59.2)	9 (75)	5 (62.5)	56 (61.5)
31-40	15 (21.1)	3 (25)	1 (12.5)	19 (20.9)
Gravida				
Primigravia	43 (60.6)	4 (33.3)	5 (62.5)	52 (57.1)
Multigravida	28 (39.4)	8 (66.7)	3 (37.5)	39 (42.9)
**Family history of hypertension**xs				
Yes	25 (35.2)	2 (16.7)	2 (25)	29 (31.9)
No	46 (64.8)	10 (83.3)	6 (75)	62 (68.1)

Out of the patients with PIH, nearly one thirds 24 (26.7%) had experienced headache, followed by dizziness 14 (15.4%) during their prenatal phases but very few 1 (1%) had symptoms such as dyspnea and chest pain (Table 2).

**Table 2:** Symptoms experienced by patients with PIH (n = 91).

^*^Symptoms	Pregnancy induced hypertension			Total n (%)
	GTN n (%)(n = 71)	Pre-eclampsia n (%) (n=12)	Eclampsia n (%) (n = 8)	
Headache	12 (16.9)	10 (83.3)	2 (25)	24 (26.4)
Epigastric pain	1 (1.4)	1 (8.3)	4 (50)	6 (6.6)
Chest pain/dyspnea	0 (0)	1 (8.3)	0 (0)	1 (1.1)
Dizziness	8 (11.3)	4 (33.3)	2 (25)	14 (15.4)

* Multiple responses

Our study showed that nearly two third 63 (69.2%) of the women with PIH had delivered by LSCS of which nearly three quarters 49 (77.8%) required emergency LSCS. Major cause for this abrupt surgery was found to be fetal distress 33 (52.4%) followed by slow progress of delivery or failed induction 21 (33.3%) deteriorating the maternal condition requiring prompt management. For eclampsia, all cases 5 (100%) were prepared for operation just to subside the maternal blood pressure (Table 3).

**Table 3:** Delivery details of patients with PIH (n = 91).

Variables	**Pregnancy induced hypertension**				Total n (%)
	GTN n (%) (n=71)	Preeclampsia n (%) (n=12)	Eclampsia n (%) (n=8)	
**Delivery type**				
SVD	23 (32.4)	2 (16.7)	3 (37.5)	28 (30.8)
LSCS	48 (67.6)	10 (83.3)	5 (62.5)	63 (69.2)
**Type of LSCS (n = 63)**	n = 48	n = 10	n = 5	
Elective LSCS	12 (25)	2 (20)	0 (0)	14 (22.2)
Emergency LSCS	36 (75)	8 (80)	5 (100)	49 (77.8)
**Reason for LSCS (n = 63)**	n = 48	n = 10	n = 5	
High Blood pressure	2 (4.2)	2 (20)	5 (100)	9 (14.3)
Meconium stain liquor/fetal distress	27 (56.2)	6 (60)	0 (0)	33 (52.4)
Slow progress/failed induction	19 (39.6)	2 (20)	0 (0)	21 (33.3)

Regarding fetal outcome before birth, most commonly seen was Non-reassuring CTG 39 (42.9%) followed by IUGR 35 (38.5%). Total IUFD was 2 (2.2%) which was seen in GTN cases. After birth, 14 (15.4%) had APGAR score <7 within 1 minute and 3 (3.3%) had <7 within 5 minutes, which is highly seen among Eclamptic mother. Most commonly found fetal outcome after birth was LBW 35 (38.4%) followed by Prematurity 24 (26.4%) whereas 33 (36.3%) were shifted to NICU for further management. One neonatal death was seen in Eclamptic case. Other fetal outcomes are presented in Table 4.

**Table 4:** Fetal status among the patients with PIH (n = 91).

^*^Fetal status	Pregnancy induced hypertension			Total
	GTN n (%) (n=71)	Preeclampsia n (%) (n=12)	Eclampsia n (%) (n=8)	n (%)
**Before birth**					
Oligohydramnious	3 (4.23)	2 (16.67)	1 (12.5)	6 (6.5)
Fetal tachycardia	14 (19.71)	0 (0)	3 (37.5)	17 (18.7)
Non-reassuring CTG	30 (42.25)	5 (41.67)	4 (50.0)	39 (42.9)
IUGR	24 (33.80)	7 (58.33)	4 (50.0)	35 (38.5)
IUFD	2 (2.82)	0 (0)	0 (0)	2 (2.2)
**After birth**				
**APGAR score at 1min**					
<7	11 (15.5)	1 (8.3)	2 (25.0)	14 (15.4)
≥7	60 (84.5)	11 (91.7)	6 (75.0)	77 (84.6)
**APGAR score at 5 mins**				
<7	2 (2.8)	0 (0)	1 (12.5)	3 (3.3)
≥7	69 (97.2)	12 (100)	7 (87.5)	88 (96.7)
Prematurity	15 (21.12)	5 (41.67)	4 (50.0)	24(26.4)
Low birth weight (LBW)	24 (33.80)	7 (58.33)	4 (50.0)	35 (38.4)
Meconium aspiration	11 (15.5)	6 (50)	4 (50.0)	21 (23.1)
Admission to NICU	22 (30.99)	6 (50)	5 (62.5)	33 (36.3)
Neonatal death	0 (0)	0 (0)	1 (12.5)	1 (1.1)

* *Multiple responses

The most common maternal outcome was preterm labor 24 (26.4%) followed by postpartum hemorrhage 8 (8.8%) and abruption placenta 2 (2.2%). There was no mortality recorded due to this morbid condition. Other maternal outcomes in different types of PIH are presented in Table 5.

**Table 5:** Maternal details after delivery of patients with PIH (n = 91).

After Delivery	Pregnancy induced hypertension			Total
	GTN n (%) (n = 71)	Preeclampsia n (%) (n = 12)	Eclampsia n (%) (n = 8)	n (%)
PPH	6 (8.4)	1 (8.3)	1 (12.5)	8 (8.8)
Preterm	15 (21.1)	5 (41.6)	4 (50)	24 (26.4)
DIC	1 (1.4)	0 (0)	0 (0)	1 (1.1)
Placental abruption	2 (2.8)	0 (0)	0 (0)	2 (2.2)

## DISCUSSION

The magnitude of PIH in our study was 6.43% which seems to be quite higher than the finding from the similar study done in Ethiopia where it was 2.4%.^8^ Here, among the women with PIH, 71 (78.1%) had gestational hypertension, 12 (13.2%) had pre-eclampsia and 8 (8.7%) had eclampsia. Similar study done by Kolluru V, et al.^7^ has contrast finding showing 27.3% cases of preeclampsia but the incidence of eclampsia was somewhat similar that showed 11.1%. This study showed that most of the women 56 (61.5%) who had PIH were of age group 21-30yrs. The result was similar to the result of the cross-sectional prospective study conducted on Paropakar Maternity and Women's Hospital,^9^ Nepal on 2018 which showed highest incidence (32.5%) in both age groups of 20-24yrs and 25-29yrs. Similar finding is seen in studies done by Singh, et al.^10^ and Aabidha, et al.^11^ in India. Another cross-sectional study of World Health Organization (WHO) also showed highest incidence of preeclampsia and eclampsia in age group of 20-24 yrs.^12^ Gestational hypertension (60.6%), and eclampsia (62.5%) were common in primigravida followed by second gravid and onwards but preeclampsia (66.7%)was more common in multigravida. The result was similar to the study done by Thakur & Dangal that also showed highest incidence of GTN and eclampsia in primigravida but in contrast preeclampsia was also common in primigravida as compared to other subsequent gravidas.^9^Other studies by Hernandez, et al.^13^ and Andrews, et al.^14^ also shows the contrasting finding of higher incidence of preeclampsia in primigravida.

Our study reveals headache (26.4%) and dizziness (15.4%) as the most common symptoms experienced by women with PIH. Epigastric pain was experienced by half (50%) of the women with eclampsia and chest pain with dyspnea was experienced only by pre-eclamptic women. In a study done in Nepal, headache was also the most common symptom (47.5%) among women with PIH followed by blurring of vision (31.25%) and epigastric pain (31.25%).^9^ Headache was found as most common symptom in the women with pre-eclampsia and eclampsia in other studies also.^2,10^ This study reveals that most common fetal complication before birth was non-reassuring CTG 39 (42.9%) and among all PIH cases, IUGR was seen on 38.5% cases (GTN 33.8%, pre-eclampsia 58.33% and eclampsia 50%, cases) which was similar to the findingsof the study done by Thakur & Dangal.^9^ In our study, total IUFD was 2 (2.2%) which was seen in only in GTN cases but similar study shows contrast finding where IUFD was seen among only pre-eclamptic cases (6.25%).^9^ Most common fetal complication after birth was LBW (38.4%) in PIH, (GTN 33.8%, pre-eclampsia 58.33% and eclampsia 50% cases), followed by prematurity (26.4%) (GTN 12.12%, pre-eclampsia 41.67% and eclampsia 50%, cases), and 33 (36.3%) were shifted to NICU for further management (GTN 30.99%, Preeclampsia 50.0% and Eclampsia 62.5%, cases) which are similar to the results of other studies.^9,15^ Among the eclamptic cases, 12.5% had neonatal death which was the total neonatal death among 91 PIH cases and it is contrasting to the study which reveals that the total neonatal deaths was only found among pre-eclampsia case (12.5%).^9^ This differences may be due to limited sample size.

The most common maternal complication in our study is found to be preterm delivery 26.4% (gestational hypertension 21.1%, preeclampsia 41.6%, and eclampsia 50%) followed by postpartum hemorrhage 8.8%. Moreover, similar results were seen in the study by Thakur & Dangal^9^ revealing preterm labor 25% as the most common maternal complication with 5.56% in gestational hypertension, 43.75% in preeclampsia and 33.33% in eclampsia respectively.^16^

Our study was conducted in one of the referral hospital, conducted within a limited duration of time with limited sample which causes over estimation of pregnancy outcome in PIH. Only PIH cases were analyzed without control groups leading inadequate comparison among different types of PIH. Further case control studies can be conducted for better comparison between and within groups.

## CONCLUSIONS

Our study shows that the prevalence of pregnancy induced hypertension is higher than compared to that of other developing countries with significant negative outcomes both seen in mothers and newborn which remains a major concern for health workers to plan for adequate supplies and manpower to manage such cases beforehand. Majority of the cases requiring operative surgeries and well equipped NICUs can be seen among PIH cases and further comparative studies are required to confirm various factors described here to be associated with PIH.
